# Antidepressant-induced mania in panic disorder: a single-case study of clinical and functional connectivity characteristics

**DOI:** 10.3389/fpsyt.2023.1205126

**Published:** 2023-05-25

**Authors:** Byung-Hoon Kim, Seung-Hyun Kim, Changsu Han, Hyun-Ghang Jeong, Moon-Soo Lee, Junhyung Kim

**Affiliations:** ^1^Department of Psychiatry, Yonsei University College of Medicine, Seoul, Republic of Korea; ^2^Institute of Behavioral Sciences in Medicine, Yonsei University College of Medicine, Seoul, Republic of Korea; ^3^Department of Psychiatry, Korea University Guro Hospital, Korea University College of Medicine, Seoul, Republic of Korea; ^4^Department of Life Sciences, Korea University, Seoul, Republic of Korea

**Keywords:** panic disorder, bipolar disorder, resting-state functional connectivity, amygdala, default mode network, frontoparietal network, biomarkers, antidepressant induced mania

## Abstract

**Background:**

Mental health issues, including panic disorder (PD), are prevalent and often co-occur with anxiety and bipolar disorders. While panic disorder is characterized by unexpected panic attacks, and its treatment often involves antidepressants, there is a 20–40% risk of inducing mania (antidepressant-induced mania) during treatment, making it crucial to understand mania risk factors. However, research on clinical and neurological characteristics of patients with anxiety disorders who develop mania is limited.

**Methods:**

In this single case study, we conducted a larger prospective study on panic disorder, comparing baseline data between one patient who developed mania (PD-manic) and others who did not (PD-NM group). We enrolled 27 patients with panic disorder and 30 healthy controls (HCs) and examined alterations in amygdala-based brain connectivity using a seed-based whole-brain approach. We also performed exploratory comparisons with healthy controls using ROI-to-ROI analyses and conducted statistical inferences at a threshold of cluster-level family-wise error-corrected *p* < 0.05, with the cluster-forming threshold at the voxel level of uncorrected *p* < 0.001.

**Results:**

The patient with PD-mania showed lower connectivity in brain regions related to the default mode network (left precuneous cortex, maximum z-value within the cluster = −6.99) and frontoparietal network (right middle frontal gyrus, maximum z-value within the cluster = −7.38; two regions in left supramarginal gyrus, maximum z-value within the cluster = −5.02 and −5.86), and higher in brain regions associated with visual processing network (right lingual gyrus, maximum z-value within the cluster = 7.86; right lateral occipital cortex, maximum z-value within the cluster = 8.09; right medial temporal gyrus, maximum z-value within the cluster = 8.16) in the patient with PD-mania compared to the PD-NM group. One significantly identified cluster, the left medial temporal gyrus (maximum z-value within the cluster = 5.82), presented higher resting-state functional connectivity with the right amygdala. Additionally, ROI-to-ROI analysis revealed that significant clusters between PD-manic and PD-NM groups differed from HCs in the PD-manic group but not in the PD-NM group.

**Conclusion:**

Here, we demonstrate altered amygdala-DMN and amygdala-FPN connectivity in the PD-manic patient, as reported in bipolar disorder (hypo) manic episodes. Our study suggests that amygdala-based resting-state functional connectivity could serve as a potential biomarker for antidepressant-induced mania in panic disorder patients. Our findings provide an advance in understanding the neurological basis of antidepressant-induced mania, but further research with larger cohorts and more cases is necessary for a broader perspective on this issue.

## 1. Introduction

Panic disorder (PD) is a common mental disorder often encountered by primary care physicians (1). The hallmark of PD is repeated unexpected panic attacks (2). Its lifetime prevalence in the general population ranges from 3.3 to 7% (3). Among the pharmacological agents, antidepressants, including serotonin-norepinephrine reuptake inhibitors and selective serotonin reuptake inhibitors, are usually considered the first-line treatment for PD (4). However, the use of antidepressants in patients with newly diagnosed PD requires caution, as antidepressants may have a 20–40% risk of inducing mania (5). Antidepressant-induced mania has been proposed as mania or hypomania that develops within 8 week of starting a new conventional antidepressant (6, 7). The unintentional occurrence of mania can have detrimental effects on patients. Therefore, understanding the risk factors for mania in patients with panic attacks is helpful for managing PD.

The comorbidity of anxiety and bipolar disorder (BD) is prevalent (8). In clinical and epidemiological studies on BD, extremely high rates of comorbid anxiety disorders have been reported (9–11). However, there is a lack of knowledge regarding the clinical features and neuroimaging results of mania occurrence in anxiety disorders, including PD (12). According to the Diagnostic and Statistical Manual of mental disorders-5, PD can be diagnosed concurrently with BD (2); however, treatment for panic attacks and related anxiety symptoms differs when there is a comorbid diagnosis of BD (10). Therefore, examining the clinical features and neuroimaging findings before mania in cases where mania develops during treatment for an anxiety disorder without a history of BD is clinically significant.

To our knowledge, research on the clinical and neurological characteristics of patients with anxiety disorders who develop mania after therapy is lacking. Significant advancements in understanding the pathogenesis, making accurate diagnoses, and investigating novel therapeutics for neuropsychiatric disorders can be achieved through the combined efforts of clinical, experimental, and computational medicine (13, 14). There are several findings regarding the clinical characteristics of patients who develop mania during their first depressive episode (15). Some studies have suggested that mania is more likely to occur in depression with prominent anxiety (16). A previous study suggested that comorbid anxiety is a subthreshold bipolarity in depressive episodes (17). However, due to the high comorbidity of depression and anxiety in clinical practice (18) and the limitations of clinical assessment of behavior by interviews (19), there is no consensus on which clinical features suggest subthreshold bipolarity in PD.

Efforts have been made to identify biomarkers for BD prediction using neuroimaging (20). Among the various biomarkers, studies have investigated abnormalities in physiological responses, such as bradycardia, as well as molecular pathways related to mitochondrial function and inflammation (21–24). With advances in techniques, functional magnetic resonance imaging (fMRI), which is the imaging of patterns of brain activity and connections, has been extensively utilized in studies on most psychiatric disorders including BD and PD (25, 26). Resting-state fMRI (rs-fMRI) analyzes variations in blood oxygen level-dependent (BOLD) signals throughout the brain in the absence of emotional or cognitive task involvement (27). rs-fMRI studies have also been conducted extensively in bipolar, bipolar I, and bipolar II disorders and in depressive (hypo) manic, which are acute states of patients with BD (28, 29). Neuroimaging studies have presented several neurobiological hypotheses regarding BD, most of which assume ventral and dorsal brain stream disturbance (20, 28). The ventrolateral prefrontal cortex, dorsal anterior cingulate cortex, dorsolateral prefrontal cortex, and hippocampus comprise the dorsal network, which mediates cognitive processing and executive functions. The ventral neural stream regulates implicit emotions via the amygdala, insula, ventral anterior cingulate cortex, ventral striatum, and ventromedial prefrontal cortex (20, 25, 28).

Alterations in amygdala-based connectivity have been consistently reported in the remission state (euthymic mood) of patients with BD compared with healthy controls (HCs) (30). However, among amygdala-based connectivities, the default mode network (DMN)-related connectivity, depending on mood state, has been reported to decrease in the (hypo) manic episodes (31) and increase in the euthymic or depressive episodes (32). Increased activity in the amygdala-insula, a key region of the DMN, has also been reported to be positively correlated with anxiety (33). Additionally, the medial prefrontal cortex (MPFC), a key component of the DMN, has been demonstrated to be a major contributor to fear inhibition (i.e., fear extinction) in human fear learning models, which are essential in understanding PD (34). Given the risk and impact of mania in treating patients with PD, investigating neuroimaging data before mania onset in patients with PD-mania would be important to determine whether it can be used as a biomarker for prediction of BD. However, to our knowledge, there is insufficient information on this topic.

This report discusses the case of a young man diagnosed with PD with no history of BD who developed mania after treatment with antidepressants. This patient participated in a prospective study related to PD; therefore, we obtained rs-fMRI images before mania onset. In addition, rs-fMRI images of patients with PD who had not developed mania at a follow-up of more than 4 months and of HCs in that study were also acquired. Therefore, this report discusses the differences in the clinical features and amygdala-based resting-state functional connectivity (rsFC) in patients who experience mania during treatment. We believe that this study will support healthcare providers in considering neuroimaging for predicting mania in patients with PD.

## 2. Materials and methods

### 2.1. Study design and participants

The current study is a single case study of antidepressant-induced mania in the longitudinal follow-up of a prospective study aimed to develop a predictive model of treatment response and prognosis in patients with PD using brain images combined with virtual reality-based anxiety behavior evaluation systems (35). In this cross-sectional analysis, baseline data were compared between the single case with mania and other patients without mania.

Patients with PD were recruited through outpatient clinics and public advertisements. A psychiatrist interviewed all patients using the Mini-International Neuropsychiatric Interview to screen for psychiatric illnesses and substance use (36). The eligibility criteria for patients with PD were the same as those used in the feasibility study of the virtual reality-based anxiety behavior evaluation systems (35). The local ethics committee of Korea University Guro Hospital approved the study (2021GR0321). Each participant completed a written informed consent form after being informed of the aims, methodology, and potential risks of the study. The study was conducted in accordance with the Declaration of Helsinki 1964.

Twenty-seven PD and 30 HCs met the eligibility criteria and participated in the study. During the study, one patient progressed to a manic episode (PD-manic patient), and the remaining 26 patients with PD did not progress to mania (PD-NM group) after at least 4 months of follow-up. All patients with PD, including PD-manic patient in the study, used serotonin-norepinephrine reuptake and selective serotonin reuptake inhibitors at baseline, according to treatment guidelines.

### 2.2. Case presentation

A 22-year-old man patient visited our psychiatric clinic in April 2022, reporting recurrent panic attacks and anticipatory anxiety since February 2022. The patient, a university student with no psychiatric history, first sought psychiatric care in February 2022 after experiencing a panic attack that manifested as palpitations, shortness of breath, chest pain, dizziness, and a sense of impending doom. He reported impairment in social functioning due to anticipatory anxiety, experienced 1–2 or more panic attacks daily, and feared being alone. After a limited therapeutic response with persistent panic attacks and anxiety for 2 months at the primary clinic, he was referred to our clinic for further evaluation.

The patients did not report any family history of any psychiatric disorder, including PD, mood disorders, or suicide attempts, was not discovered. He denied any history of BD and alcohol or benzodiazepine abuse. Table 1 presents the patient's demographic and psychological characteristics in April 2022. The patient had a Panic Disorder Severity Scale (PDSS) score of 14, indicating moderate to severe social impairment and 2-3 panic attacks per week (37, 38). Other anxiety-related assessments were mostly moderate to severe (LSAS-fear: 44, LSAS-avoidance: 40, GAD: 17, HADS-anxiety: 16). The patient reported a moderate level of depression (HADS-depression: 11) but no neurovegetative symptoms and did not meet the criteria for major depressive disorder (39, 40). Based on his medical history and the Diagnostic and Statistical Manual of mental disorders-5 (2), PD was diagnosed.

**Table 1:** Demographic and psychological characteristics of two groups (HC and PD-NM) and the patient with PD-mania.

	**HC (*****n*** = **30)**		**PD-NM (*****n*** = **26)**		***t/χ^2^***	***p*-value**	**Patient with PD-mania**
	**Mean/*n***	**SD/%**	**Mean/n**	**SD/%**			
Age (years)	33.60	9.65	34.92	10.74	−0.485	0.629	22
Sex (woman, %)	8	26.7	14	53.8	3.250	0.071	Male
Education (years)	15.23	1.55	13.69	2.43	2.870	0.006	12
PDSS	0.00	0.00	14.77	3.49	−23.208	< 0.001	14
**LSAS**							
Fear	21.53	14.49	27.62	15.07	−1.538	0.13	44
Avoidance	19.43	12.54	26.50	16.45	−1.821	0.074	40
GAD	3.67	3.44	11.04	5.47	−6.119	< 0.001	17
**HADS**							
Anxiety	4.60	2.82	12.27	4.75	−7.463	< 0.001	16
Depression	6.57	3.96	11.19	4.12	−4.278	< 0.001	11

The statistical value was the *t*-value in an independent *t*-test for continuous data and χ*2* in the chi-square test for categorical data. SD, standard deviation; PDSS, Panic Disorder Severity Scale; LSAS, Liebowitz Social Anxiety Scale; GAD, Generalized Anxiety Disorder-7; HADS, Hospital Anxiety Depression Scale.

For the first 2 months, the patient was prescribed medications, including an adequate dose of escitalopram (20 mg/day), desvenlafaxine (100 mg/day), and aripiprazole (5 mg/day), as augmentation treatment. Alprazolam (0.25 mg) and propranolol (10 mg) were prescribed up to three times daily for symptomatic relief of panic attacks and anxiety. After 8 weeks of treatment (June 2022), the patient's frequency of panic attacks decreased to 1–2 times per week, with moderate improvement in the associated anticipatory anxiety, avoidance behaviors, and related social behavior problems. However, he was rated as moderately ill, with a panic disorder severity scale score of 14.

For the patient's symptoms associated with PD, desvenlafaxine was increased from the previous dose to 200 mg/day in June 2022, and aripiprazole was increased to 7 mg/day 4 weeks later. In July 2022, the treatment effect was unsatisfactory, with no further improvement in panic attacks and anxiety (panic disorder severity scale score of 14) and worsening depression with neurovegetative symptoms. In August 2022, increased impulsivity, overspending, and polydipsia were reported, and a manic episode was considered, with a score of 18 on the Young Mania Rating Scale (YMRS). Escitalopram and desvenlafaxine were tapered off and escalated to lithium (900 mg/day) and quetiapine XR (600 mg/day) for 5 weekweeks. The patient returned to a euthymic state with YMRS scores of 12 and 6 in September 2022 and October 2022, respectively. During the 2 months, the patient did not report any panic attacks.

The patient participated in a prospective study conducted in our psychiatric clinic in June 2021; therefore, we obtained fMRI images produced when he was diagnosed with and treated for PD before his manic episode.

### 2.3. Psychological assessments

The same assessments as those in the VRABES feasibility study were performed at baseline before the fMRI. PDSS was used as an interview measure of PD severity (37), and the Liebowitz Social Anxiety Scale Self-Report Version (LSAS) (41), Generalized Anxiety Disorder Scale (42), and Hospital Anxiety and Depression Scale (HADS) were used to examine anxiety-related traits (43). For patients with mania, YMRS data were collected in an outpatient setting, as clinically indicated (44).

### 2.4. Imaging data acquisition

A 3 T Philips Ingenia scanner (Philips Healthcare, Best, Netherlands) was used to collect the images. Coronal anatomical images were acquired using a 3D T1-weighted (T1w) fast gradient echo sequence (repetition time (TR) = 4.6 ms, echo time (TE) = 9.9 ms, 220 slices, slice thickness = 1 mm, and field of view (FOV) = 224 × 224 × 224). For the functional images, a T2^*^-weighted (BOLD) image was obtained (TR = 2,000 ms, TE = 22 ms, 38 slices, slice thickness = 3 mm, FOV = 210 × 210 × 216, and voxel size = 3 × 3 × 3 mm). Images were acquired in the transverse orientation. The participants were advised to relax, stay awake, keep their eyes open, and focus on the cross symbol displayed on the screen throughout the scanning process.

### 2.5. Preprocessing

The results included in this manuscript are from the preprocessing performed using fMRIPrep 21.0.2 (45), which is based on Nipype 1.8.1 (46, 47).

#### 2.5.1. Anatomical data preprocessing

A total of one T1w images were discovered within the input Brain Imaging Data Structure dataset. All images were corrected for intensity non-uniformity with N4BiasFieldCorrection (48) and distributed with Advanced Normalization Tools (ANTs) 2.3.3 (RRID: SCR_004757) (49). The T1w-reference was then skull-stripped with a Nipype implementation of the antsBrainExtraction.sh workflow (from ANTs), using OASIS30ANTs as the target template. Brain tissue segmentation of the cerebrospinal fluid (CSF), white matter (WM), and gray matter was performed on brain-extracted T1w images using fast (FSL 6.0.5.1:57b01774, RRID: SCR_002823) (50). A T1w-reference map was computed after the registration of two T1w images (after intensity non-uniformity correction) using the mri_robust_template (FreeSurfer 6.0.1) (51). Volume-based spatial normalization to one standard space (MNI152NLin2009cAsym) was performed through non-linear registration with antsRegistration (ANTs 2.3.3) using brain-extracted versions of both the T1w reference and T1w template. The following template was selected for spatial normalization: *ICBM 152 Non-linear Asymmetrical template version 2009c* (52) (RRID: SCR_008796; TemplateFlow ID: MNI152NLin2009cAsym).

#### 2.5.2. Functional data preprocessing

The following preprocessing was performed for each BOLD run obtained per participant (across all tasks and sessions). First, a reference volume and its skull-stripped version were generated using the custom fMRIPrep methodology. Head-motion parameters with respect to the BOLD reference (transformation matrices and six corresponding rotation and translation parameters) were estimated before spatiotemporal filtering using mcflirt (FSL 6.0.5.1:57b01774) (53). The BOLD time series (including slice-timing correction when applied) was resampled onto its original native space by applying transforms to correct for head motion. These resampled BOLD time series are called *preprocessed BOLD in original space* or just *preprocessed BOLD*. The BOLD reference was then coregistered with the T1w reference using mri_coreg (FreeSurfer), followed by flirt (FSL 6.0.5.1:57b01774) (54) with the boundary-based registration (55) cost function. Co-registration was configured with six degrees of freedom. Several confounding time series were calculated based on the *preprocessed BOLD*: framewise displacement (FD), differential variation in signals (DVARS), and three region-wise global signals. The FD was computed using two formulations: power (absolute sum of relative motions) (56) and Jenkinson (relative root-mean-square displacement between affines) (53). The FD and DVARS were calculated for each functional run using their implementations in *Nipype* (56). Three global signals were extracted from the CSF, WM, and whole-brain masks. In addition, a set of physiological regressors were extracted to allow for component-based noise correction (*CompCor*) (57). Principal components were estimated after high-pass filtering of the *preprocessed BOLD* time series (using a discrete cosine filter with a 128 s cut-off) for the two *CompCor* variants: temporal (tCompCor) and anatomical (aCompCor). The tCompCor components were calculated from the top 2% of the variable voxels within the brain mask. For aCompCor, three probabilistic masks (CSF, WM, and combined CSF+WM) were generated in anatomical space. The implementation differs from that of Behzadi et al.; instead of eroding the masks by two pixels in the BOLD space, the aCompCor masks were subtracted from a mask of pixels that likely contained a volume fraction of the gray matter. This mask was obtained by thresholding the corresponding partial volume map at 0.05, which ensured that the components were not extracted from the voxels containing a minimal fraction of gray matter. Finally, these masks were resampled into the BOLD space and binarized by thresholding at 0.99 (as in the original implementation). The components of the WM and CSF masks were calculated separately. For each CompCor decomposition, the k components with the largest singular values were retained, such that the time series of the retained components was sufficient to explain 50% of the variance across the nuisance mask (CSF, WM, combined, or temporal). The remaining components were excluded from the analysis. The head motion estimates calculated in the correction step were also placed in the corresponding confounding file. The confounded time series derived from the head motion estimates and global signals was expanded by including temporal derivatives and quadratic terms (58). Frames that exceeded a threshold of 0.5 mm FD or 1.5 standardized DVARS were annotated as motion outliers. The BOLD time series was resampled into a standard space, generating a *preprocessed* BOLD run in *MNI152NLin2009cAsym space*. A reference volume and its skull-stripped version were generated using the custom methodology of *fMRIPrep*. All resamplings were performed in a single interpolation step by composing all pertinent transformations (head-motion transform matrices, susceptibility distortion correction when available, and co-registration with anatomical and output spaces). Gridded (volumetric) resampling was performed using antsApplyTransforms (ANTs), configured with Lanczos interpolation to minimize the smoothing effects of other kernels (59). Non-gridded (surface) resampling was performed using mri_vol2surf (FreeSurfer).

### 2.6. Seed-based functional connectivity

In line with our hypothesis that alterations in amygdala-based brain connectivity would appear between the PD-manic patient and PD-NM group prior to a manic episode, we conducted a seed-based, whole-brain analysis using two regions of interest (ROIs): the right and left amygdala. The automated anatomical labeling (AAL2) atlas was employed to define these ROIs (60). Anatomical atlases, such as AAL, have been shown to yield reliable and valid results (61), and recent studies on amygdala-based brain connectivity in panic disorder have also utilized the AAL2 atlas (62, 63). Temporal correlations between the BOLD signals in the left and right amygdala seed regions (AAL41 and AAL42, respectively) were computed using CONN toolbox (64). Pearson's bivariate correlation analysis was performed to calculate the connections between the seeds and other voxels in the whole brain. The resulting values were then converted to z-scores using the Fisher's r-to-z transformation. In the seconds level random effect analysis, a generalized linear model analysis of covariance was conducted to examine between-group (PD-NM group vs. PD-manic patient) differences in FC patterns, controlling for the effects of age, sex, and years of education. Statistical inferences to identify brain regions revealing significant differences were performed at the threshold of cluster-level family-wise error-corrected *p* (*p*_FWE_) < 0.05, with the cluster-forming threshold at the voxel level of uncorrected *p* < 0.001.

### 2.7. Exploratory comparisons with HCs

The main hypothesis focuses on differences between the anxiety disorder group, who did not develop mania, and patients who did; however, exploratory comparisons between HCs and the non-manic anxiety group in seed-based functional connectivity were also performed. To evaluate the degree to which observed differences between the PD-NM and PD-manic groups reflect aberrant brain functioning, ROI-to-ROI analyses were performed using both amygdalae (AAL41 & 42) and identified brain regions in the seed-based analysis as seed ROIs. Fisher's z-transformed correlations were calculated to indicate the connectivity strength between each pair of ROIs. Between-group differences (PD-NM vs. HC) were studied using analysis of covariance, controlling for age, sex, and years of education. ROI-to-ROI results were reported as significant if *p* < 0.05 andROI-level false-discovery rate corrected (*p*_*FDR*_).

### 2.8. Statistical analysis for demographic and psychological assessments

Statistical analyses of the demographic and psychological characteristics between the PD-NM and HC groups were conducted using a software package (IBM SPSS Statistics v28.0, IBM Corporation, Armonk, NY, USA). Demographics and psychological evaluations of the PD-NM and HC groups were compared using an independent *t*-test for continuous data and a chi-square test for categorical data. *p* < 0.05 was considered statistically significant.

## 3. Results

### 3.1. Demographic and psychological assessments

The demographic features and assessment scores of the PD-NM, HC, and PD-manic groups are presented in Table 1. The distributions of age, sex, and marital status between the two groups (PD-NM vs. HC) did not vary significantly. The PD-NM group had more years of education than the HC group. Except for the LSAS-fear and LSAS-avoidance subscale scores, psychological characteristics associated with anxiety differed significantly. The patient in the PD-manic group was relatively younger and had fewer years of education than the recruited patients and HCs. Scores on the LSAS-SR and anxiety subscales of the Generalized Anxiety Disorder Scale and HADS were relatively higher than the mean for the PD-NM group. This was a single case; thus, no statistical analysis could be performed. However, age, years of education, panic disorder severity scale, LSAS, Generalized Anxiety Disorder Scale, and HADS scores were all within two standard deviations of the distribution in the PD-NM group.

### 3.2. Neuroimaging results

**Figure 1:**
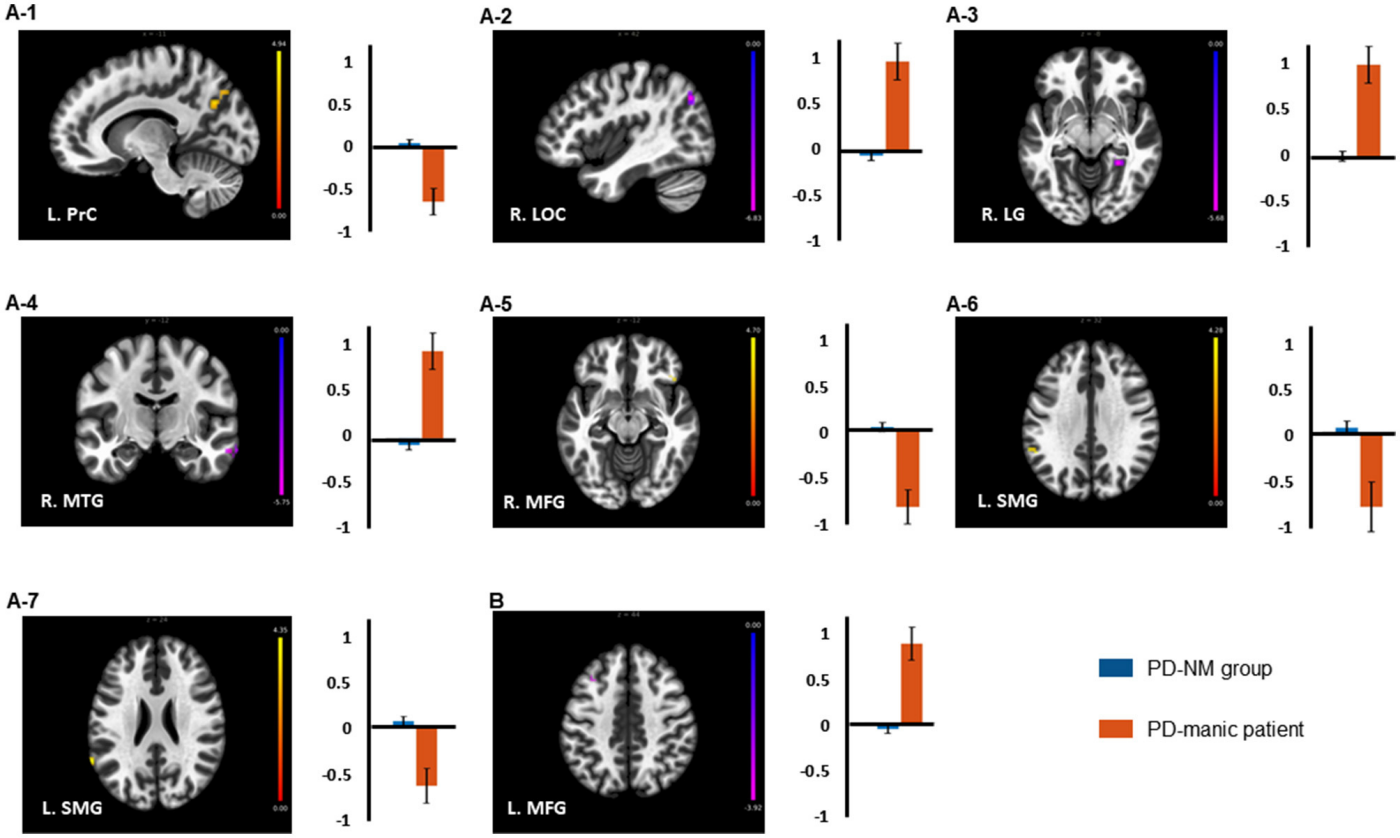
Brain regions revealed a significant difference in resting-state functional connectivity with **(A)** left and **(B)** right amygdala seed between the PD-NM and PD-manic groups. Error bars indicate the standard errors of the mean. L, left; R, right; PrC, precuneous cortex; MFG, Middle frontal gyrus; SMG, supramarginal Gyrus; LOC, lateral occipital cortex; LG, right lingual gyrus; MTG, middle temporal gyrus.

After adjusting for the potential effects of age, sex, and years of education, seven clusters with the left amygdala and one with the right amygdala in which the PD-NM group and PD-manic patient differed were identified (Table 2). Compared with the PD-NM group, the PD-manic patient demonstrated lower rsFC between the left amygdala and left precuneus cortex (PrC), right middle frontal gyrus (MFG), and two left supramarginal gyri (SMG), and higher rsFC between the left amygdala and right lateral occipital cortex (LOC), right lingual gyrus (LG), and right middle temporal gyrus (MTG). One identified cluster, the left MTG, presented higher rsFC with the right amygdala (Figure 1).

**Table 2:** Results of the seed-based functional connectivity analysis between the PD-NM group and the patient with PD-mania.

**Seed**	**Target**	**N_vox_**	**peak MNI (mm)**			**Z_max_**
			**x**	**y**	**z**	
L. Amygdala	L. Precuneous cortex	74	−14	−80	40	−6.99
	R. Lateral Occipital Cortex	50	42	−68	36	8.09
	R. Lingual gyrus	36	28	−44	−8	7.86
	R. Middle Temporal Gyrus	36	62	−12	−20	8.16
	R. Middle Frontal Gyrus	30	46	22	−8	−7.38
	L. Supramarginal Gyrus	28	−60	−44	32	−5.02
	L. Supramarginal Gyrus	24	−62	−52	24	−5.86
R. Amygdala R	L. Middle Frontal Gyrus	26	−36	22	44	5.82

MNI, Montreal Neurological Institute; N_vox_, number of voxels; Z_max_, maximum z-value within the cluster; L, left; R, right.

The relative HC and PD-NM groups revealed no suprathreshold differences in ROI-to-ROI analysis, considering both the amygdala (AAL41 & 42) and identified brain regions in the seed-based analysis as seed ROIs. However, PD-manic patients revealed lower rsFC from the left amygdala in the ROI of the two ROI in left SMG (*t*_51_ = −3.36, *p*_*FDR*_ = 0.001; *t*_51_ = −3.88, *p*_*FDR*_ < 0.001), right FOC (*t*_51_ = −6.45, *p*_*FDR*_ < 0.001), and left PrC (*t*_51_ = −3.74, *p*_*FDR*_ = 0.001) and higher rsFC in the right LOC (*t*_51_ = 5.95, *p*_*FDR*_ < 0.001), right MTG (*t*_51_ = 5.37, *p*_*FDR*__<_ 0.001), right LG (*t*_51_ = 6.94, *p*_*FDR*__<_ 0.001), and left MTG (*t*_51_ = 4.73, *p*_*FDR*_ 0.001) compared to the HC group.

In summary, the primary differences between the PD-manic patient and the PD-NM group were predominantly observed in the left amygdala-based rsFC. Additionally, an exploratory comparison analysis of the significant clusters revealed that the ROI-to-ROI connectivity between these clusters was significant only when comparing the healthy control (HC) group to the PD-manic patient, and not when comparing the HC group to the PD-NM group.

## 4. Discussion

We investigated the differences in amygdala-based rsFC between the patient with PD who developed mania during treatment and those who did not. Our findings indicate that, in the case of antidepressant-induced mania, left amygdala-based rsFC was lower in the left PrC, right MFG, and left SMG, and higher in the right LOC, right LG, and right MTG when compared to PD patients without mania. Additionally, right amygdala-based rsFC exhibited a stronger association with the left MTG in the PD-manic patient than in the PD-NM group. Furthermore, connectivity associated with significant clusters between the PD-manic patient and PD-NM groups differed from that of the HCs only in the patient with the PD-manic patient and not in the PD-NM group. The implications of these results are discussed below.

### 4.1. Functional connectivity between the amygdala and DMN

We identified reduced L. amygdala–L. PrC connectivity in the PD-manic group compared with the PD-NM group. The PrC is an important region associated with the DMN (65). Changes in functional connectivity between amygdala and DMN have been reported to vary by mood state; decrease in manic or hypomanic mood episodes (31) and increase in depressive episodes (32). The HADS-depression subscale scores of the PD-manic patient and the PD-NM group suggest that both the PD-NM group and the PD-manic patient were experiencing mild depression even though they did not meet the diagnostic criteria for major depressive disorder, which is consistent with findings from general clinical studies (66). Furthermore, although statistical analysis was not possible due to sample size, characteristics related to anxiety and depression in the PD-manic patient were within the 1 SD distribution of the PD-NM group. Consequently, it is notable that the increased rsFC between amygdala-DMN, reported in BD for (hypo)manic mood rather than depression or euthymic mood, was observed in the patient with PD-mania.

In a euthymic state or depressive episode, there is increased functional connectivity between the amygdala and the DMN (28, 32). Additionally, increased rsFC between the amygdala and the DMN has also been reported in patients with PD (53). However, reductions in fractional anisotropy have been observed in several white matter tracts in the bipolar group, including the cingulum bundle connecting the amygdala to the posterior cingulate cortex (PCC), a key region of the DMN (67). Moreover, the previous finding that reduced structural connectivity between the amygdala and DMN was observed in both BD patients and unaffected first-degree relatives suggests that decreased rsFC between the amygdala and DMN could be specific to BD (68). Consequently, the reduced L. amygdala–L. PrC in patients with PD before antidepressant treatment may be a more specific biomarker for the risk of antidepressant-induced mania.

### 4.2. Functional connectivity between the amygdala and frontoparietal network (FPN)

Reduced functional connectivity between the L. amygdala, R. MFG, and L. SMG was observed in the PD-manic group compared with the PD-NM group. In contrast, the functional connectivity between the R. amygdala-L. MFG was increased in the PD-manic group compared to the PD-NM group. Additionally, this trend of results was consistent in the exploratory comparison between the HC group and the PD-manic patient, while no significant difference was found between the PD-NM group and the HC group. The SMG and MFG are crucial nodes of the FPN (69). Alterations in rsFC between the amygdala and FPN have been reported in patients with BD (70). These findings were also replicated in a recent study which reported decreased connectivity between the amygdala and inferior frontal regions associated with the FPN in BD with (hypo) mania (28). In patients with PD, the FC between the amygdala and FPN was also decreased when exposed to emotional facial stimuli (71). Moreover, a previous study investigating rsFC following the process of fear extinction, a key component of exposure therapy used to treat panic disorder (72), reported a positive correlation between amygdala-FPN connectivity and the magnitude of fear extinction recall in healthy participants. In contrast, patients with panic disorder exhibited weaker amygdala-FPN connectivity (73). Although the comparison with healthy controls in each disease showed the same direction, to our knowledge, no studies comparing the two disease groups and no studies observing decreased rsFC in patients with PD compared to healthy controls have been identified. Therefore, our finding of decreased connectivity between the L. amygdala and FPN-related clusters in the PD-manic patient compared to the PD-NM group presents a novel result and suggests, for the first time, the possibility that relatively increased amygdala-FPN connectivity in patients with PD may reflect susceptibility to antidepressant-induced mania. Furthermore, our results, which revealed no difference in the exploratory comparison between the PD-NM and HC groups, also support the possibility that these connections could be clinically helpful.

As decreased connectivity between the amygdala and the FPN has been reported in both BD and PD compared to HC, our finding of a significant difference in the PD-manic patient needs to be viewed in the context of clinical presentation. Decreased amygdala-FPN connectivity has been linked to response inhibition (74), which may contribute to disinhibited behavior in patients with BD and their first-degree relatives (75). Moreover, previous studies concerning patients with PD suggested that decreased FC between the amygdala and FPN may play a role in the impaired fear extinction process observed in panic disorder (73). Thus, the lack of significant differences in the PD-NM group from the HC group in our exploratory analysis suggests that the alteration of rsFC between the amygdala and FPN in bipolar may differ from that in PD. It would be clinically valuable if these differences in connectivity could be confirmed by clinically observable differences in behavior, such as abnormalities in response inhibition or fear extinction reported in previous studies. However, larger studies focusing on subregions of the amygdala and FPN and anxiety behaviors are needed to examine these differences directly.

To our knowledge, there have been no reports on the asymmetric nature of the left and right amygdala-based rsFC in patients with BD. A previous study investigating the asymmetry of the left and right amygdala-based rsFC in healthy individuals reported distinct connectivity patterns and a more prominent right amygdala-based rsFC (76). Furthermore, decreases in amygdala-based rsFC in anxiety-related disorders, such as obsessive-compulsive and social anxiety disorders, have been reported only on the left side (77, 78). In task-based fMRI studies, the right amygdala is reportedly more involved in the automatic processing of environmental stimuli, and the left amygdala is more Involved in the continuous assessment of potential threats (79–82). Therefore, the increase in the right amygdala-based rsFC may reflect an increased response to external stimuli rather than anxiety. Increased response to external stimuli is also a repeatedly reported clinical feature in patients with BD (83). Therefore, the increased rsFC between the right amygdala and left MFG in the PD-manic group before a manic episode is an interesting finding that could reflect the clinical features of patients with BD. However, as we identified data from a single case, caution should be exercised when interpreting this previously unidentified finding.

### 4.3. Functional connectivity between the amygdala and visual processing networks

The PD-manic patient revealed stronger rsFC between the L. amygdala–R. LG, R, MTG, R.LOC and R. amygdala–L. MTG than the PD-NM group. The LG, MTG, and LOC are known to be involved in visual processing (84, 85). Previous findings related to the rsFC of these regions have not been investigated in patients with BD or PD. The connectivity of the visual processing network and the amygdala has been investigated in studies utilizing the visual identification of facial expressions in emotional tasks rather than in the resting state (86, 87). These findings may reflect problems with emotional processing (88). Given that problems with emotional processes in patients with BD are also seen in residual symptoms or euthymic states (89, 90)our results may reflect these traits in patients with BD. However, this interpretation should be taken cautiously, as we demonstrated functional connectivity in only one patient. Further studies on amygdala connectivity in the visual processing network are required.

### 4.4. Limitations and future directions

Our study had several limitations that constrain its interpretations. First, this study characterized one unexpected case in a longitudinal study; therefore, the altered amygdala-based functional connectivity discovered in this study cannot be generalized to patients with PD concerning antidepressant induced mania. However, we conducted an exploratory comparison with the HC group to support the specificity of our results and obtained results consistent with our hypotheses. Second, we did not control for the type and dose of medication. As different classes of antidepressants vary in the risk associated with antidepressant induced mania, this may be a confounding factor; thus, our results should be interpreted with caution (91). However, the patients in this study were prescribed either serotonin-norepinephrine reuptake inhibitors or selective serotonin reuptake inhibitors at baseline, which have been reported to have a relatively similar risk of antidepressant-induced mania (91). Third, the study was not designed for antidepressant induced mania; therefore, information such as YMRS scores at baseline was unavailable. The eligibility criteria ensured that participants did not experience mania before participation; however, information on the clinical features associated with mania was limited. Fourth, as this is a single-case study and the analysis was focused on the amygdala to reduce the problem of multiple comparisons, the results are not necessarily informative about overall brain connectivity. To identify biomarkers associated with antidepressant-induced mania, a multicenter cohort study with a large enough number of cases to achieve statistical significance is needed. Given the comorbidity of anxiety and depression, multimodality studies that include comprehensive clinical features and genetic information should be conducted.

## 5. Conclusion

According to our findings, altered rsFC between the amygdala-DMN and amygdala-FPN, which has been reported in (hypo)manic episodes of BD, was observed both in patients with PD and HCs without mania. Additionally, differences in rsFC related to the visual processing network were identified, which have not been reported in previous studies. This study provides preliminary evidence and considerations for the future use of amygdala-based rsFC as a biomarker of antidepressant-induced mania in treating patients with PD. Further studies with larger cohorts and different cases are required to verify clinical relevance of our findings. However, given the possible practical benefits of our discovery, we believe that this study will excite both professionals and scholars.

## Permission to reuse and copyright

The boilerplate text of “2.5 Preprocessing” was automatically generated by fMRIPrep with the express intention that users should copy and paste this text into their manuscripts unchanged. It was released under the CC0 license.

## Data availability statement

The raw data supporting the conclusions of this article will be made available by the authors, without undue reservation.

## Ethics statement

The studies involving human participants were reviewed and approved by the local Ethics Committee of Korea University Guro Hospital. The patients/participants provided their written informed consent to participate in this study.

## Author contributions

Conceptualization, formal analysis, and writing—original draft preparation: B-HK and JK. Participant evaluation and data acquisition: S-HK, CH, H-GJ, and M-SL. Writing—review and editing and supervision: JK. All authors contributed to the article and approved the submitted manuscript.
